# Givinostat for Becker muscular dystrophy: A randomized, placebo-controlled, double-blind study

**DOI:** 10.3389/fneur.2023.1095121

**Published:** 2023-01-30

**Authors:** Giacomo P. Comi, Erik H. Niks, Krista Vandenborne, Claudia M. Cinnante, Hermien E. Kan, Rebecca J. Willcocks, Daniele Velardo, Francesca Magri, Michela Ripolone, Jules J. van Benthem, Nienke M. van de Velde, Simone Nava, Laura Ambrosoli, Sara Cazzaniga, Paolo U. Bettica

**Affiliations:** ^1^Neuromuscular and Rare Diseases Unit, Department of Neuroscience, Fondazione IRCCS Ca' Granda Ospedale Maggiore Policlinico, Milan, Italy; ^2^Department of Pathophysiology and Transplantation, Dino Ferrari Center, University of Milan, Milan, Italy; ^3^Department of Neurology, Leiden University Medical Center, Leiden, Netherlands; ^4^Duchenne Center Netherlands, Netherlands; ^5^ImagingDMD, University of Florida, Gainesville, FL, United States; ^6^Radiology Department, Istituto Auxologico Italiano, IRCCS, Milan, Italy; ^7^Department of Radiology, C.J. Gorter MRI Center, Leiden University Medical Center, Leiden, Netherlands; ^8^Neurology Unit, Fondazione IRCCS Ca' Granda Ospedale Maggiore Policlinico, Milan, Italy; ^9^Department of Orthopedics, Rehabilitation and Physiotherapy, Leiden University Medical Center, Leiden, Netherlands; ^10^OPIS srl, Milan, Italy; ^11^Italfarmaco SpA, Milan, Italy

**Keywords:** Becker muscular dystrophy, therapy, disease progression, fibrosis, magnetic resonance imaging (MRI)

## Abstract

**Objective:**

No treatments are approved for Becker muscular dystrophy (BMD). This study investigated the efficacy and safety of givinostat, a histone deacetylase pan-inhibitor, in adults with BMD.

**Methods:**

Males aged 18–65 years with a diagnosis of BMD confirmed by genetic testing were randomized 2:1 to 12 months treatment with givinostat or placebo. The primary objective was to demonstrate statistical superiority of givinostat over placebo for mean change from baseline in total fibrosis after 12 months. Secondary efficacy endpoints included other histological parameters, magnetic resonance imaging and spectroscopy (MRI and MRS) measures, and functional evaluations.

**Results:**

Of 51 patients enrolled, 44 completed treatment. At baseline, there was greater disease involvement in the placebo group than givinostat, based on total fibrosis (mean 30.8 vs. 22.8%) and functional endpoints. Mean total fibrosis did not change from baseline in either group, and the two groups did not differ at Month 12 (least squares mean [LSM] difference 1.04%; *p* = 0.8282). Secondary histology parameters, MRS, and functional evaluations were consistent with the primary. MRI fat fraction in whole thigh and quadriceps did not change from baseline in the givinostat group, but values increased with placebo, with LSM givinostat–placebo differences at Month 12 of −1.35% (*p* = 0.0149) and −1.96% (*p* = 0.0022), respectively. Adverse events, most mild or moderate, were reported by 88.2% and 52.9% patients receiving givinostat and placebo.

**Conclusion:**

The study failed to achieve the primary endpoint. However, there was a potential signal from the MRI assessments suggesting givinostat could prevent (or slow down) BMD disease progression.

## 1. Introduction

Becker muscular dystrophy (BMD) is a heterogeneous muscle disease, with substantial variability in age of onset and clinical presentation (1). In the early stages BMD involves active myonecrosis and regeneration; later in the disease course chronic myopathic changes are more likely, including increased skeletal muscle fiber size variability, fibrosis, and fat replacement of muscle tissue (2, 3), with selective muscle hypertrophy (4, 5). Initial symptoms may include cramping and reduced endurance during exercise (6). This is followed by gradual muscle weakness in the hips, pelvis, thighs, and shoulders, leading to walking on toes with lumbar lordosis and early loss of ambulation, although some patients are able to remain ambulatory even into their 60s (6). No treatments are specifically approved for BMD.

BMD is caused by in-frame mutations in the dystrophin gene (although with exceptions), resulting in a reduced amount or truncated size of the dystrophin protein (7). Dystrophin assembles with other proteins to form the dystrophin-associated protein complex (DAPC), which plays a critical role in stabilizing the plasma membrane of striated muscle by linking the actin cytoskeleton to the extracellular matrix. Neuronal nitric oxide synthase (nNOS) is an important component of the DAPC. Nitric oxide produced by nNOS inactivates histone deacetylase (HDAC) 2 *via* S-nitrosylation of a cysteine residue (8). This mechanism is dysfunctional in dystrophic muscle, leading to aberrantly upregulated HDAC activity (8). A potential target of therapy for BMD is therefore HDAC inhibition. Indeed, in a dystrophin-deficient mouse model inhibition of HDAC activity led to functional and morphological muscle recovery (9).

In this manuscript, we report the results of a study that investigated the efficacy and safety of givinostat, a HDAC pan-inhibitor, in adults with BMD.

## 2. Materials and methods

This was a Phase II, randomized, double-blind, placebo-controlled study that aimed to evaluate the effects of givinostat on micro- and macroscopic muscle morphology and function. The study was conducted in two centers: University of Milan, Italy, and Leiden University Medical Center, the Netherlands, with the University of Florida (ImagingDMD) serving as the central data management and processing center for magnetic resonance imaging (MRI) and spectroscopy (MRS). Following a four-week screening period, patients were randomized to receive givinostat or placebo for 12 months, attending study visits every 2 weeks for the first 2 months, then every 12 weeks for the remainder of the study.

Eligible patients were males aged 18–65 years, inclusive, with a clinical diagnosis of BMD confirmed by genetic testing (based on patient records), and who were able to walk between 200 and 450 m in the 6 min walk test (6MWT). If patients were receiving a systemic corticosteroid, angiotensin converting enzyme inhibitor, or α- or β-adrenergic receptor blocker they were expected to have no significant change in dose or regimen immediately prior to the start of study treatment. Among the reasons for exclusion were: Use of any pharmacologic treatment, other than corticosteroids, or surgery in the 3 months prior to study entry that might have an effect on muscle strength or function; symptomatic cardiomyopathy or heart failure (New York Heart Association Class III or IV) or left ventricular ejection fraction <50% at screening or with heart transplant; and contraindications to muscle biopsy or magnetic resonance scans. All patients provided written informed consent prior to any study-related procedure. Full inclusion and exclusion criteria are listed in the Supplementary material. The study was approved by an independent ethics committee for each institution, and was performed in accordance with the principles of the Declaration of Helsinki and the International Conference on Harmonization notes for guidance on Good Clinical Practice (ICH/CPMP/135/95). The study was registered at ClinicalTrials.gov (NCT03238235).

During the screening period and after 12 months, chemical shift encoded MRI and MRS of the right lower leg, thigh and gluteus maximus, and an open muscle biopsy of the brachial biceps were performed (see Supplementary methods for detailed methodology and the specific muscles included in each muscle group, and Supplementary Figure 1 for an example MRI image). Patients were randomized on entry to the study, such that 50% had their right arm biopsied at baseline and the left arm at the end of the study; the other 50% had their left arm biopsied at baseline and the right arm at study end. In addition, patients undertook a series of timed-function tests (rise from floor, run/walk 10 m, and climb four standard steps) and a 6MWT (10). They then completed the Motor Function Measure (MFM) (11–13), followed by bilateral strength measures (knee extension and elbow flexion) using hand-held myometry (microFET Dynamometer, Hoggan Scientific LLC, Salt Lake City, UT, USA). The functional and strength assessments were evaluated by qualified functional evaluators (all physiotherapists), with the timed-function tests and 6MWT standardized by a study-specific manual. Safety was assessed throughout the study, in terms of adverse events, hematology, blood chemistry, physical examination and electrocardiogram parameters, and lung function. Selected baseline data, and the correlations between these data, have been published in a previous manuscript (14).

The protocol was amended four times. The main changes in the first amendment were the addition of MRI evaluations of the lower leg and an increase in maximum age from 60 to 65 years. Amendment 2 resulted from a blinded evaluation of data from the first 21 patients, in which 11 patients required dose reduction due to thrombocytopenia, and four patients had high plasma triglyceride levels. The givinostat starting dose was therefore reduced (from the “high dose” to the “low dose,” see Supplementary material), and additional safety dosing rules were introduced (see the “Interventions” Section). Amendment 3 added an interim analysis of baseline characteristics. Amendment 4 changed the primary endpoint as a result of the pre-planned blinded interim analysis to check the sample size (see the sample size Section). The primary endpoint was originally change from baseline in fiber cross-sectional area (CSA) after 12 months of treatment. However, the mean CSA of the brachial biceps fibers in the first 20 baseline biopsies was similar to age-matched CSA in healthy adults. It was therefore deemed unlikely that givinostat could increase fiber CSA. Given fibro-adipose replacement is a hallmark of BMD (2), total fibrosis was considered a more indicative primary outcome measure. The revised sample size calculation increased the required number of patients from 48 to 51.

### 2.1. Interventions

Patients were randomized 2:1 *via* an interactive web response system to receive givinostat or matching placebo, stratified by concomitant steroid use at baseline (yes or no). Patients, investigators, and site and sponsor staff were blinded to treatment assignment. Given platelet count reductions are observed after administration of givinostat, personnel who performed the various efficacy analyses were different from those who recorded the safety results.

Givinostat (10 mg/mL) or placebo oral suspension were administered using a graduated dosing syringe as two daily doses (morning and evening) after a meal. The starting dose was selected according to body weight as shown in Supplementary Table 1, and rules were pre-specified for treatment to be permanently discontinued or temporarily interrupted (see Supplementary methods). In the case of treatment interruption, the dose was reduced by 20% once platelets, white blood cells, hemoglobin and/or triglycerides were normal, or diarrhea was mild. In addition, if a patient had at least two consecutive platelet counts ≤ 150 × 10^9^/L that did not meet the stopping criteria, the dosage was reduced by 20% of the current dose.

### 2.2. Outcomes

The primary objective was to demonstrate statistical superiority of givinostat over placebo in terms of the mean change from baseline in total fibrosis after 12 months of treatment. Secondary efficacy endpoints included change from baseline after 12 months of treatment in: other histological parameters (% muscle fiber area [MFA], % adipose tissue, % fibers with nuclear centralizations, % regenerative fibers, fiber total CSA, fiber size variability, and total dystrophin); MRI measures of muscle fat fraction, MRI CSA and contractile area in the gluteus maximus, thigh and lower leg muscles; MRS fat fraction of the vastus lateralis and soleus; MFM (total and component); timed-function tests (time to climb four standard steps, time to walk/run 10 m, and time to rise from the floor); 6MWT; and muscle strength of the knee extension and elbow flexion measured by hand-held myometry. Additional details on the histology, MRI, MRS, and functional endpoints are in the Supplementary material.

### 2.3. Sample size and statistical methods

It was calculated that 48 patients with evaluable baseline biopsies (32 and 16 receiving givinostat and placebo, respectively), would provide 80% power to test the null hypothesis of no treatment effect on total fibrosis vs. the alternative hypothesis that the treatment effect was ≥9%, using a two-sided *t*-test with alpha level of 5% and assuming a common standard deviation (SD) of 10% (based on blinded interim data from the first 20 patients). Allowing for 5% of patients with unevaluable biopsies, the total number of patients to be randomized was 51 (34 and 17, respectively).

Since the blinded data at the time of the sample size re-estimation indicated non-normal distribution of the primary efficacy variable, mean fibrosis at baseline and Month 12 were log transformed prior to analysis, and then back-transformed. An analysis of covariance (ANCOVA) model was fitted to the data with the difference between log Month 12 and log baseline values as the dependent variable, log baseline value as covariate, and treatment and concomitant steroid use at baseline as independent class variables, and the results were presented as least squares means (LSM). The secondary histology, MRI and MRS endpoints were analyzed using a similar method to the primary objective, although without log transformation. The functional endpoint objectives were analyzed using a mixed model for repeated measures, with fixed effect class terms included for treatment, visit, visit by treatment interaction, and concomitant steroid use at baseline, and the baseline value included as a covariate. An unstructured covariance matrix was used to model the within-patient error.

The intent-to-treat (ITT) set was used for all efficacy analyses, and comprised all patients randomized to treatment and who received at least one dose of study medication. The safety set comprised all patients who received at least one dose of study medication, and was used for all safety analyses.

## 3. Results

### 3.1. Participants

**Figure 1:**
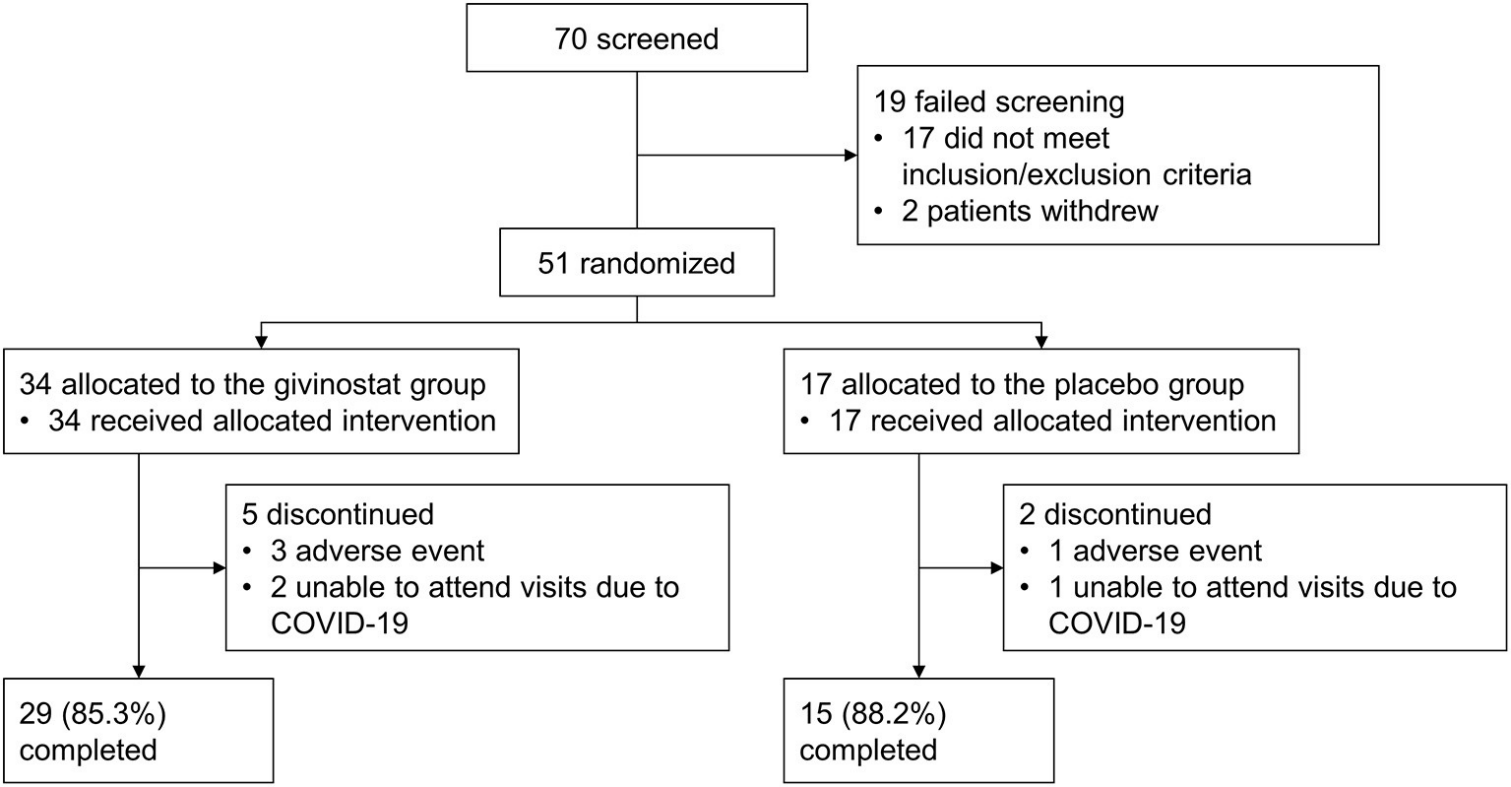
Patient disposition.

The study was conducted between January 2018 and March 2021. Of 70 patients screened, 51 were enrolled, 44 of whom completed study treatment (Figure 1). The ITT and safety sets were identical. Patients in the givinostat group were on average slightly younger than those in the placebo group, had a shorter time since diagnosis, and were more likely to have a mutation upstream of exon 45 (Table 1).

**Table 1:** Patient baseline demographics and disease characteristics.

	**Givinostat** ** (*N* = 34)**	**Placebo** ** (*N* = 17)**
Age (years), mean (SD); range	36.5 (11.56); 19–61	39.2 (9.84); 24–58
Sex, male, *n* (%)	34 (100)	17 (100)
Time from diagnosis^*^ to informed consent signature (years), mean (SD); range	11.7 (7.43); 0.2–26.2	15.6 (9.97); 0.1–27.7
**Mutation type**, ***n*** **(%)**		
Duplication	1 (2.9)	0
Deletion	32 (94.1)	16 (94.1)
Point mutation	1 (2.9)	1 (5.9)
**Mutated exon category**, ***n*** **(%)**		
Exon 45	20 (58.8)	14 (82.4)
Downstream from exon 45	2 (5.9)	1 (5.9)
Upstream of exon 45	12 (35.3)	2 (11.8)
**Concomitant use of steroids**, ***n*** **(%)**	2 (5.9)	1 (5.9)
Deflazacort	2 (5.9)	0
Prednisone	0	1 (5.9)

* Based on genetic testing.

### 3.2. Outcomes

#### 3.2.1. Histology

For the primary endpoint, total fibrosis, the mean value at baseline was higher in the placebo group than the givinostat group, suggesting a greater degree of disease involvement (Table 2). Over the duration of the study, mean values did not change from baseline in either group (with 95% CIs of the log LSM values including 0), and there was no difference between the two groups in the Month 12 assessment. Most of the secondary histology parameters were consistent with the primary endpoint, in that baseline values were generally worse in the placebo group, with either no or minimal change from baseline over the study duration, and no differences between the groups for the Month 12 assessment (Table 2, Supplementary Table 2).

**Table 2:** Histology parameters at baseline and Month 12.

**Endpoint treatment group**	**Baseline, mean (SD)**	**Month 12**	
		**Change from baseline**, **LSM (95% CI)**	**Givinostat–placebo difference**, **LSM (95% CI);** ***p*****-value**
**Total fibrosis, %**			
Givinostat	22.8 (11.36)	0.98 (0.69, 1.40)^*^	1.04 (0.74, 1.46); 0.8282
Placebo	30.8 (11.33)	0.95 (0.60, 1.49)^*^	
**Muscle fiber area, %**			
Givinostat	75.1 (14.07)	0.42 (−10.20, 11.04)	2.45 (−7.87, 12.77); 0.6340
Placebo	65.7 (14.74)	−2.03 (−16.25, 12.20)	
**Adipose tissue, %**			
Givinostat	1.4 (3.62)	1.09 (0.70, 1.72)	0.87 (0.58, 1.30); 0.4893
Placebo	2.6 (4.67)	1.25 (0.71, 2.22)	
**Fiber with nuclear centralizations, %**			
Givinostat	16.9 (7.96)	1.22 (0.83, 1.78)	0.77 (0.55, 1.06); 0.1055
Placebo	20.9 (11.77)	1.59 (1.01, 2.51)	
**Regenerative fibers, %**			
Givinostat	6.4 (8.94)	1.95 (0.97, 3.92)	0.65 (0.35, 1.19); 0.1562
Placebo	3.6 (4.08)	3.02 (1.28, 7.11)	
**Total CSA**, μ**m**^2^			
Givinostat	5,615 (1,921.7)	643 (−603, 1,888)	−573 (−1,691, 546); 0.3070
Placebo	4,741 (2,263.7)	1,215 (−360, 2,790)	
**CSA type I**, μ**m**^2^			
Givinostat	5,340 (2,251.9)	1.29 (0.93, 1.79)	1.01 (0.75, 1.36); 0.9493
Placebo	4,899 (3,998.4)	1.28 (0.85, 1.93)	
**CSA type II**, μ**m**^2^			
Givinostat	5,925 (2,399.2)	−25 (−1,438, 1,388)	−619 (−1,872, 634); 0.3240
Placebo	5,178 (1,812.0)	594 (−1,178, 2,366)	
**Fiber size variability**			
Givinostat	4,751 (1,939.9)	−820 (−1,971, 332)	−304 (−1,296, 688); 0.5389
Placebo	4,928 (3,131.2)	−515 (−1,920, 889)	

* The log LSM changes from baseline in total fibrosis were −0.017 (95% CI −0.367, 0.333) and −0.054 (−0.507, 0.399) for givinostat and placebo, respectively. LSM, least squares mean; CSA, cross-sectional area.

#### 3.2.2. MRI/MRS

**Figure 2:**
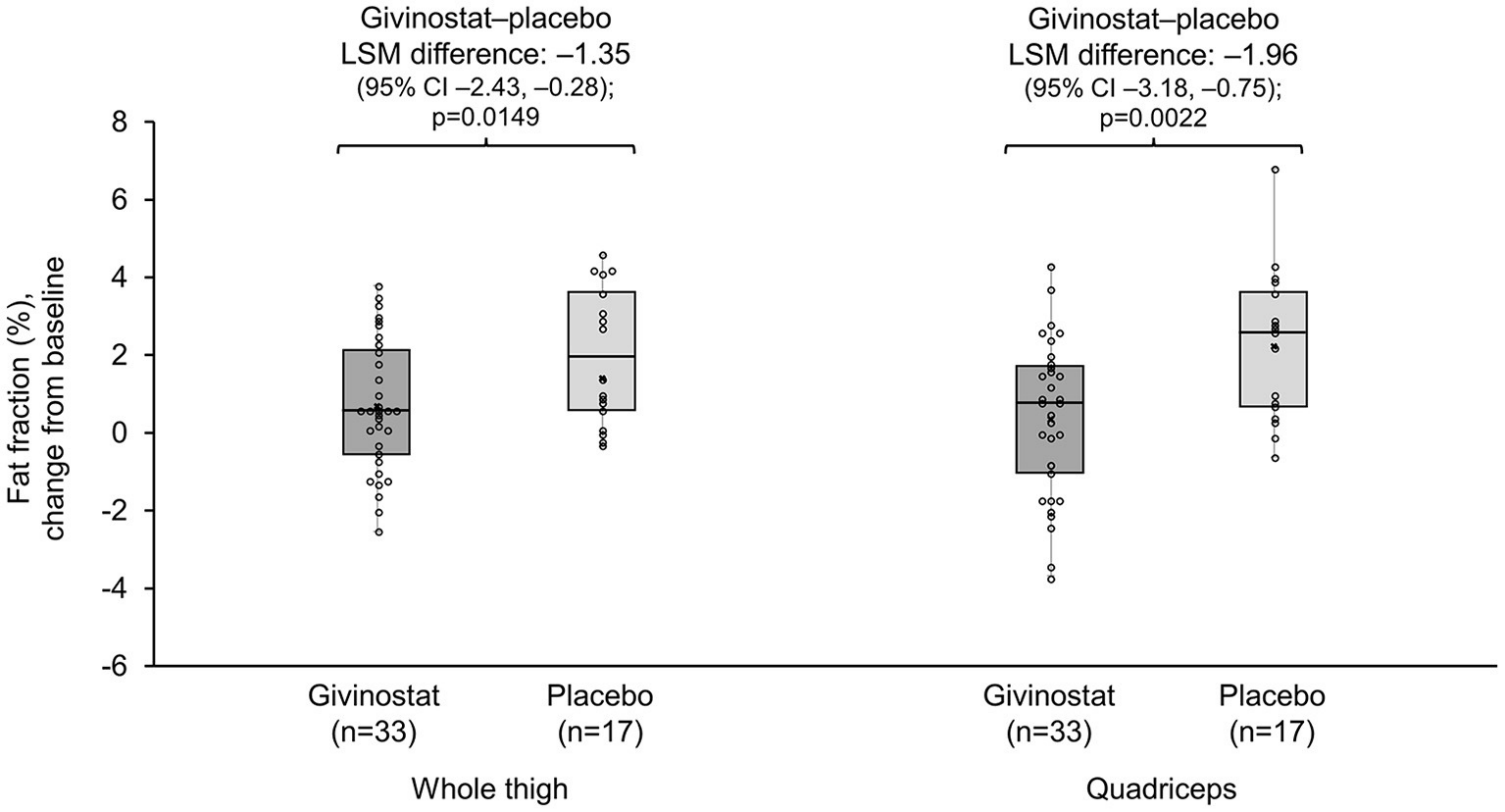
MRI whole thigh and quadriceps fat fractions change from baseline at Month 12. Data plotted are mean change from baseline (mid-point), interquartile range (box), and range (whisker), with individual patient data overlaid. Mean (SD) whole thigh percentages at baseline were 56.0 (13.78) and 63.5 (13.23) for givinostat and placebo, respectively; baseline quadriceps percentages were 54.7 (15.45) and 60.3 (14.43), respectively. MRI, magnetic resonance imaging; LSM, least squares mean.

For the MRI fat fraction endpoints, values did not change from baseline over the study duration in the givinostat group, but there were increases (i.e., worsening) from baseline at Month 12 in the placebo group for fat fraction in both the whole thigh and quadriceps, with resultant LSM differences between the two groups of −1.35% (*p* = 0.0149) and −1.96% (*p* = 0.0022), respectively (Figure 2, Table 3). The other muscle groups showed similar directions of changes, but there were no givinostat–placebo differences (Table 3).

**Table 3:** MRI and MRS fat fractions at baseline and Month 12.

**Endpoint treatment group**	**Baseline, mean (SD)**	**Month 12**	
		**Change from baseline**, **LSM (95% CI)**	**Givinostat–placebo difference**, **LSM (95% CI);** ***p*****-value**
**MRI, %**			
**Whole thigh** ^*^			
Givinostat	56.0 (13.78)	0.64 (−0.33, 1.61)	−1.35 (−2.43, −0.28); 0.0149
Placebo	63.5 (13.23)	2.00 (0.81, 3.18)	
**Quadriceps** ^*^			
Givinostat	54.7 (15.45)	0.61 (−0.51, 1.73)	−1.96 (−3.18, −0.75); 0.0022
Placebo	60.3 (14.43)	2.57 (1.23, 3.92)	
**Medial thigh** ^*^			
Givinostat	46.9 (12.63)	1.01 (0.98, 1.04)	0.97 (0.94, 1.01); 0.1165
Placebo	55.8 (15.87)	1.04 (1.00, 1.08)	
**Hamstrings** ^*^			
Givinostat	66.5 (19.95)	0.88 (−0.63, 2.38)	−0.58 (−2.24, 1.08); 0.4869
Placebo	74.4 (15.92)	1.46 (−0.40, 3.31)	
**Triceps surae** ^*^			
Givinostat	36.0 (12.91)	−0.28 (−2.86, 2.31)	−1.59 (−3.47, 0.29); 0.0939
Placebo	37.2 (10.61)	1.32 (−1.69, 4.32)	
**Gluteus maximus**			
Givinostat	62.8 (13.04)	0.58 (−0.51, 1.68)	−0.89 (−2.13, 0.36); 0.1579
Placebo	71.0 (7.66)	1.47 (0.11, 2.83)	
**MRS, %**			
**Vastus lateralis**			
Givinostat	55.7 (19.70)	1.02 (0.95, 1.09)	0.95 (0.89, 1.03); 0.1991
Placebo	55.5 (17.13)	1.07 (0.98, 1.16)	
**Soleus**			
Givinostat	26.7 (17.34)	−4.29 (−6.13, −2.45)	−0.27 (−2.22, 1.69); 0.7849
Placebo	22.2 (16.69)	−4.02 (−6.40, −1.64)	

* See the Supplementary material for details of the muscles included in these groups. MRI, magnetic resonance imaging; MRS, magnetic resonance spectroscopy; LSM, least squares mean.

The MRI contractile area endpoints were broadly consistent with the MRI fat fraction endpoints, with trends to decreases (i.e., worsening) from baseline in the placebo group, but no change with givinostat (Supplementary Table 3). The two groups differed in terms of the whole thigh contractile area assessment (LSM difference 1.37 cm^2^; *p* = 0.0375), and approached a difference in the quadriceps assessment (0.63 cm^2^; *p* = 0.0528). There were no differences between groups for the MRI CSA endpoints (Supplementary Table 3), or for the two MRS fat fraction endpoints (Table 3).

#### 3.2.3. Functional endpoints

As with the histology endpoints, baseline functional endpoint values were consistent with greater disease involvement in the placebo group, with lower MFM score, longer times required to complete the timed-function tests, a shorter distance covered in the 6MWT, and lower values in the hand-held myometry assessments (Table 4, Supplementary Table 4). Changes over the duration of the study in these parameters were small, and there were no differences between the two groups at Month 12.

**Table 4:** Functional endpoints at baseline and Month 12.

**Endpoint treatment group**	**Baseline, mean (SD)**	**Month 12**	
		**Change from baseline, LSM (95% CI)**	**Givinostat–placebo difference, LSM (95% CI);** ***p*****-value**
**Motor function measurement scores**			
**Standing and transfers (D1)**			
Givinostat	52.1 (12.14)	0.96 (0.92, 1.01)	1.06 (1.00, 1.14); 0.0602
Placebo	42.4 (11.40)	0.91 (0.85, 0.97)	
**Axial and proximal motor function (D2)**			
Givinostat	98.8 (2.29)	1.00 (0.99, 1.00)	1.00 (0.99, 1.01); 0.5906
Placebo	96.4 (4.69)	0.99 (0.98, 1.00)	
**Distal motor function (D3)**			
Givinostat	98.6 (2.76)	1.00 (0.99, 1.01)	1.00 (0.99, 1.01); 0.7799
Placebo	98.3 (2.89)	1.00 (0.99, 1.01)	
**Total score**			
Givinostat	79.8 (5.76)	0.99 (0.98, 1.00)	1.01 (1.00, 1.03); 0.1116
Placebo	74.9 (5.78)	0.97 (0.96, 0.99)	
**Timed-function tests, sec**			
**Time to climb four standard steps**			
Givinostat	9.3 (16.02)	0.87 (0.71, 1.05)	0.98 (0.72, 1.33); 0.8914
Placebo	11.0 (14.83)	0.88 (0.67, 1.16)	
**Time to walk/run 10 m**			
Givinostat	9.1 (5.66)	1.01 (0.91, 1.13)	0.94 (0.79, 1.11); 0.4346
Placebo	9.3 (3.03)	1.08 (0.93, 1.26)	
**Time to rise from floor**			
Givinostat	8.1 (3.82)	1.39 (−0.97, 3.75)	0.62 (−3.51, 4.75); 0.7629
Placebo	11.4 (8.50)	0.77 (−3.01, 4.56)	
**6 min walk test, m**			
Givinostat	365.5 (69.61)	0.94 (0.89, 1.00)	0.99 (0.90, 1.09); 0.8106
Placebo	333.9 (67.91)	0.96 (0.88, 1.04)	

LSM, least squares mean.

### 3.3. Safety

A total of 17 patients commenced the study on high-dose givinostat, 17 on low-dose givinostat, and 17 on placebo. Subsequently, 26 patients (82.4%) in the givinostat group had their dose reduced due to an adverse event (either decreased platelet count or hypertriglyceridemia). Three patients (8.8%) in the givinostat group and one patient (5.9%) in the placebo group permanently discontinued treatment due to adverse events, one as a result of decreased platelet count (in the givinostat group) and three for hypertriglyceridemia (two in the givinostat group, and one in the placebo group).

All of the patients receiving high-dose givinostat experienced at least one adverse event, most commonly decreased platelet counts and diarrhea (Table 5). However, the majority were mild or moderate in severity, with none either serious or fatal. A lower proportion of patients receiving low-dose givinostat experienced adverse events (76.5%)—and again the majority were mild or moderate in severity, with none either serious or fatal.

**Table 5:** Adverse events by treatment received at baseline, overall and most common preferred terms (≥3 patients in any group for adverse events and treatment-related adverse events; ≥2 patients in any group for adverse events leading to treatment interruption or withdrawal, and severe adverse events).

**Number (%) of patients**	**Givinostat**		**Placebo** ** (*N* = 17)**
	**High dose** **(*****N*** = **17)**	**Low dose** **(*****N*** = **17)**	
**Adverse event**	17 (100)	13 (76.5)	9 (52.9)
Abdominal pain upper	3 (17.6)	0	1 (5.9)
Blood triglycerides increased	4 (23.5)	0	1 (5.9)
Diarrhea	10 (58.8)	6 (35.3)	0
Hypertriglyceridemia	4 (23.5)	6 (35.3)	1 (5.9)
Platelet count decreased	15 (88.2)	5 (29.4)	0
**Treatment-related** **adverse event**	17 (100)	12 (70.6)	4 (23.5)
Abdominal pain upper	3 (17.6)	0	1 (5.9)
Blood triglycerides increased	4 (23.5)	0	1 (5.9)
Diarrhea	10 (58.8)	6 (35.3)	0
Hypertriglyceridemia	3 (17.6)	6 (35.3)	1 (5.9)
Platelet count decreased	15 (88.2)	5 (29.4)	0
**Adverse event** **leading to** **treatment** **interruption**	7 (41.2)	4 (23.5)	1 (5.9)
Hypertriglyceridemia	2 (11.8)	2 (11.8)	0
Platelet count decreased	4 (23.5)	0	0
**Adverse event** **leading to** **treatment** **withdrawal**	1 (5.9)	2 (11.8)	1 (5.9)
**Severe adverse** **event**	3 (17.6)	2 (11.8)	0
Hypertriglyceridemia	1 (5.9)	2 (11.8)	0
**Treatment-related** **severe adverse** **event**	2 (11.8)	2 (11.8)	0
Hypertriglyceridemia	1 (5.9)	2 (11.8)	0
**Severe adverse** **event leading to** **treatment** **interruption**	2 (11.8)	1 (5.9)	0
**Severe adverse** **event leading to** **treatment** **withdrawal**	0	1 (5.9)	0
**Serious adverse** **event**	0	0	0
**Fatal adverse event**	0	0	0

The mean platelet count was lower at baseline in the givinostat group than the placebo group (Supplementary Table 5). There was a reduction from baseline in the mean value at Month 12 in the givinostat group, and no change in the placebo group (although with high variability around the mean in both groups), with 36.4% of patients in the givinostat group having a shift from normal to low platelet counts during the study (compared with none in the placebo group); 52.9% of patients in the givinostat group required a reduction in dose due to decreased platelet count. Mean triglyceride levels increased from baseline to Month 12 in the givinostat group, with no change in the placebo group; 50.0% of patients in the givinostat group had a shift from normal levels at baseline to high levels at some point during the study (Supplementary Table 5). There were only minor changes in other hematology and blood chemistry values, with no relevant changes in physical examination or electrocardiogram parameters or lung function.

## 4. Discussion

This was the first clinical trial to evaluate the effects of givinostat in BMD. The study aimed to evaluate givinostat effects on muscle morphology (by histology and magnetic resonance), and on muscle function and strength, as well as evaluating tolerability. The primary objective was to demonstrate superiority of givinostat over placebo in terms of the mean change from baseline in total fibrosis after 12 months of treatment. This was not met. This was also true for the other histological parameters. In contrast, whole thigh and quadriceps MRI measures showed muscle deterioration in the placebo group (increased fat replacement and decline in contractile area), but no change from baseline over the 12 month follow-up period in the givinostat group. This resulted in differences between the two groups at Month 12, providing a potential signal that the use of givinostat was associated with stabilization of disease progression.

The contrasting study results, with no difference between the two groups in the histological and functional assessments but differences in the whole thigh and quadriceps MRI assessments may, of course, indicate that givinostat is not effective in this BMD population. However, givinostat has previously demonstrated efficacy in mouse DMD models (15, 16), and has also been shown to increase the fraction of muscle tissue and to reduce the amount of fibrotic tissue in boys with DMD (17). It is generally assumed that if a molecule is effective in DMD it will also be effective in BMD, although there is no specific preclinical model of BMD, and no molecules have so far been approved in both DMD and BMD. Furthermore, although corticosteroids are standard of care in the management of DMD (18), efficacy is less clear in BMD (6). Alternative explanations of these results are the imbalance at baseline between groups in histological parameters, including fibrosis, as well as the stability of the histological parameters over time. Given that givinostat is more likely to work by slowing down muscle deterioration, the selected patient population, who did not show muscle histological deterioration, was possibly not suited to show an effect of the drug. Furthermore, the histological and functional measures may not be sensitive enough in the BMD population as endpoints, as indicated by the lack of change over 12 months. In addition, whereas MRI assesses disease involvement across a large area, biopsy captures involvement in a small fraction of the muscle. Moreover, the muscles in the upper extremities (the site of the biopsies) are less likely to show pathology in BMD than the proximal lower extremity muscles (the location of the MRI). It would therefore have been interesting to conduct MRI of the upper extremity muscles in the current study to allow a direct comparison with the biopsy results.

A number of previous studies have used MRI to evaluate muscle involvement and disease progression in BMD (19–31), with skeletal muscle fat fractions correlating with motor function (19, 21), and changes predicting functional deterioration (19). In one of these, MRI-assessed muscle fat fraction increased significantly over 24 months in the thigh [median +1.9% (−0.7–5.4), *p* = 0.01] (21). This is consistent with the change that we observed in the placebo group in our study. For a patient with BMD, the fat fraction increase over time can be described by a sigmoidal curve, similar to that which has been shown in DMD (32–34), and the baseline value can therefore predict future fat fractions (23, 35). Given these various findings, it may be useful not only to stratify patients into future studies by their functional status (the 6MWT may not be the best instrument for this), but, if MRI is to be used as an endpoint, according to their MRI fat fraction (22).

Whereas, givinostat had an effect on some of the MRI endpoints, this was not the case with either of the MRS endpoints. This is likely attributable to the large degree of tissue heterogeneity in BMD, which, as we reported previously (14), is difficult to reliably capture using single voxel MRS. In contrast, the analysis of MRI images follows the contour of the muscles and reflects a larger region of muscle tissue (14). MRS results may therefore not be representative of the whole muscle in BMD (in contrast with DMD), and the quantification of fat fraction in this population may necessitate using an image-based MRI.

Givinostat and placebo also did not differ in terms of the strength assessments, timed-function tests or 6MWT, although there was a trend to stabilization of the MFM D1 and total score. Similar to the histology results, baseline imbalances between groups, and lack of deterioration over time in the placebo group, may explain these findings. In a previous manuscript, we used baseline data from the current study to assess correlations between various parameters (14). Despite substantial heterogeneity between patients, MRI fat fractions in the whole thigh and quadriceps correlated significantly with the functional endpoints. This, together with the observations from the current study that the MRI assessments could detect progression in fat replacement in patients receiving placebo, suggests that givinostat effects on functional endpoints could be observed in a study with longer duration and with a patient population that is in a more rapid disease decline phase.

The safety profile of givinostat in the current study was consistent with previous studies in other diseases, including JAK2 positive chronic myeloproliferative neoplasms such as polycythemia vera (36–38), with adverse events being predominantly mild to moderate in severity and more frequent at the higher dose. The exception was hypertriglyceridemia, which was not tested in previous studies. This was therefore the first study to include detailed post-baseline evaluations of triglyceride levels, and it is of note that 41.2% of patients receiving placebo had high triglyceride levels at some point during the study, one of whom discontinued study treatment. Further work is therefore needed to determine the clinical relevance of these findings.

The main limitations of this study are associated with the unexpected difference in disease involvement at baseline between the two patient groups. This, together with the relatively small sample size (although appropriate for this type of study) and high inter-patient variability in the various outcome measures (typical of BMD), suggests caution in the interpretation of the overall findings. In addition, it is clear that total fibrosis is not the best primary endpoint for a study in BMD, given the minimal changes over the follow-up period (suggesting that a longer follow-up would be required to detect any changes, and even then perhaps only in patients with later-stage disease), and other measures such as MRI-based quantification of fat fraction may be better suited. Furthermore, a large number of patients did not meet the eligibility criteria (39), mainly due to the ambulatory criteria and the high ejection fraction. This will be important to consider for future protocols. The heterogeneity of BMD also contributes to the challenges of conducting studies in this disease—a range of BMD phenotypes were eligible for inclusion in the study. The key strengths of the study are that it was conducted at just two clinical sites, both of which are experienced at conducting these evaluations, with all MRS/MRI data evaluated in a single laboratory (University of Florida).

Overall, the efficacy results from the current study highlight the challenges of conducting a study in BMD, a relatively slowly-progressing disorder where the main aim is to prevent (or at least delay) disease progression, rather than cure. Although the study failed to achieve the primary endpoint, there was a potential signal from the MRI assessments that suggests givinostat could prevent (or at least slow down) disease progression in BMD, slowing fat replacement in the whole thigh and quadriceps muscles. This study also provides additional support to the use of MRI as an assessment tool in future BMD studies.

## Data availability statement

The raw data supporting the conclusions of this article are available on reasonable request from the sponsor, following submission of a valid research protocol to the corresponding author.

## Ethics statement

The studies involving human participants were reviewed and approved by an Independent Ethics Committee for each institution. The patients/participants provided their written informed consent to participate in this study.

## Author contributions

GC, EN, KV, RW, and MR: conceptualization, methodology, investigation, writing—review and editing, and visualization. CC, HK, DV, FM, JB, NV, SN, and LA: methodology, investigation, writing—review and editing, and visualization. SC and PB: conceptualization, methodology, supervision, writing—review and editing, and visualization. All authors approved the submitted version of the manuscript.
