# Supplementary material for: Givinostat for Becker muscular dystrophy: A randomized, placebo-controlled, double-blind study
Source: Front Neurol. 2023 Jan 30;14:1095121. doi: 10.3389/fneur.2023.1095121 (PMC9923355; doi:10.3389/fneur.2023.1095121)
Supplement: Supplementary file 1 [file Data_Sheet_1.PDF]

Givinostat for Becker muscular dystrophy: A randomized, placebo-controlled, double-blind study

Giacomo P Comi, Erik H Niks, Krista Vandenborne, Claudia M Cinnante, Hermien E Kan, Rebecca J Willcocks, Daniele Velardo, Francesca Magri, Michela Ripolone, Jules J van Benthem, Nienke M van de Velde, Simone Nava, Laura Ambrosoli, Sara Cazzaniga, Paolo U Bettica

Supplement

Methods

Inclusion criteria

1. Ambulant male patients 18 to 65 years old at randomization with a diagnosis of BMD confirmed by genetic testing.
2. Able and willing to give informed consent in writing.
3. Able to perform the 6MWT at screening with a minimum distance of 200 m and maximum distance of 450 m.
4. If receiving treatment with systemic corticosteroids and/or angiotensin converting enzyme (ACE) inhibitor, and/or β- or α-adrenergic receptor blocker, no significant change in dosage or dosing regimen (excluding changes related to body weight) was to be present for a minimum of 6 months prior to start of study treatment.
5. Patients had to be willing to use adequate contraception from randomization until 3 months after the last dose of study treatment, and included the following:

- True abstinence when in line with the preferred and usual lifestyle of the patient. Periodic abstinence (e.g., calendar ovulation, symptothermal, post ovulation methods) and withdrawal were not acceptable methods of contraception.
- Condom with spermicide, with the female partner using an acceptable method of contraception, such as an oral, transdermal, injectable or implanted steroid-based contraceptive, or a diaphragm or a barrier method of contraception in conjunction with spermicidal jelly such as a cervical cap with spermicide jelly.

Exclusion Criteria

1. Exposure to another investigational drug within 3 months prior to the start of study treatment.
2. Use of any pharmacological treatment, other than corticosteroids, that could have affected muscle strength or function within 3 months prior to the start of study

treatment (e.g., growth hormone). Vitamin D, calcium, and other supplements were allowed.

3. Surgery that could have affected muscle strength or function within 3 months before study entry or planned surgery at any time during the study.
4. Presence of other clinically significant disease that in the Investigator's opinion could have adversely affected the safety of the patient or could have impaired the assessment of study results.
5. A diagnosis of other uncontrolled neurological diseases or presence of relevant somatic disorders not related to BMD that could have interfered with the ability to perform the muscle function tests and/or to comply with the study protocol procedures.
6. Platelet count, white blood cell (WBC) count and hemoglobin at screening less than the lower limit of normal (LLN). If laboratory screening results were <LLN, platelet count, WBC count and hemoglobin were to be repeated once, and if again <LLN became exclusionary.
7. Symptomatic cardiomyopathy or heart failure (New York Heart Association Class III or IV) or left ventricular ejection fraction <50% at screening or with heart transplant.
8. Current liver disease or impairment, including but not limited to elevated total bilirubin (>1.5 x upper limit of normal [ULN]), unless secondary to Gilbert's disease or pattern consistent with Gilbert's disease.
9. Inadequate renal function defined by serum cystatin C >2 x ULN. If the value was >2x ULN, serum cystatin C was to be repeated once; if this again >2 x ULN it was exclusionary.
10. Positive test for hepatitis B surface antigen, hepatitis C antibody, or human immunodeficiency virus at screening.
11. Baseline QT interval corrected using Fridericia's correction (QTcF) >450 msec (as the mean of three consecutive readings 5 min apart) or history of additional risk

factors for torsades de pointes (e.g., heart failure, hypokalemia, or family history of long QT syndrome).

12. Current psychiatric illness/social situations rendering the patient unable to understand or comply with the muscle function tests and/or with the study protocol procedures.
13. Hypersensitivity to the components of the study medication.
14. Sorbitol intolerance or sorbitol malabsorption, or the hereditary form of fructose intolerance.
15. Contraindications for muscle biopsy.
16. Contraindications for MRI/MRS (e.g., claustrophobia, metal implants or seizure disorders).
17. Hypertriglyceridaemia (>1.5 x ULN). At screening, patients with hypertriglyceridaemia could be enrolled if on stable treatment and with controlled levels of triglycerides (i.e., within normal range) for at least 6 months.

Histology methodology

Muscle biopsy samples were immediately frozen in liquid nitrogen-cooled 2-methylbutane and then stored in liquid nitrogen. Serial transverse 8 µm thick cryosections were processed for routine staining with hematoxylin and eosin (H&E), and with myosin ATPase (pH 9.4). On each section, four randomly selected, non-overlapping fields were photographed at 20x magnification, using optical microscope Leica DC200 equipped with camera and IM50 image analysis software (Leica Microsystems, Wetzlar, Germany). Each muscle biopsy was blindly evaluated by two operators. All morphometric analyses were performed using ImageJ 1.51j8 (https://imagej.nih.gov/ij/download.html) and LAS 4.9.0 (Leica Application Suite) software, consistent with the methods of Peverelli and colleagues.(1) On the H&E muscle sections, centronucleated fibers were counted. Regenerating fibers were manually quantified on four additional muscle sections after immunohistochemistry with fetal myosin (anti-MYH3).

On the myosin ATPase-stained section, the cross sectional area (CSA) of type I and type II fibers was measured by manually drawing the perimeter of each fiber and by calculating the corresponding area (μm^2) using LAS version 4.9.0 software. Only transverse fibers were included in the analysis.

To determine dystrophin levels, immunoreactive bands obtained by western blot were visualized by ODYSSEY LI-COR Model 2800 and analyzed by Image J. For each patient, immunoreactive bands (dystrophin rod domain and dystrophin carboxy terminus) were normalized with internal standard (actinin).

MRI and MRS methodology

MRI and MRS scans were acquired in a 1.5T (at the Milan, Italy, center; Philips Achieva, Phillips Healthcare, Amsterdam, Netherlands) or 3T (at the Leiden, Netherlands, center; Ingenia Philips, Philips Healthcare) whole body magnet. Chemical-shift encoded MRI can accurately measure fat fraction across field strengths (2), and standardized fat and copper sulphate phantoms scanned regularly in the current study confirmed the quality of fat and water separation at both field strengths. To generate fat fraction maps, multi-echo axial gradient echo images were acquired in the thigh and calf (TR = 430 msec or greater; TE = 4.61, 6.91, 9.21 msec [1.5T], 4.61, 5.76, 6.91 msec [3T]) (3). Slices were chosen to include the origin of the short head of the biceps femoris, the insertion of the popliteus, and the top of the head of the femur, which served as the landmarks for the lower leg, thigh, and gluteus maximus, respectively (Supplementary Fig 1). Single-voxel ^{1}H-MR spectroscopy data were acquired from the belly of the vastus lateralis muscle and the soleus muscle using stimulated echo acquisition mode (TR=3000; TR=108) as previously described (4).

Supplementary Figure 1. Example MRI image.

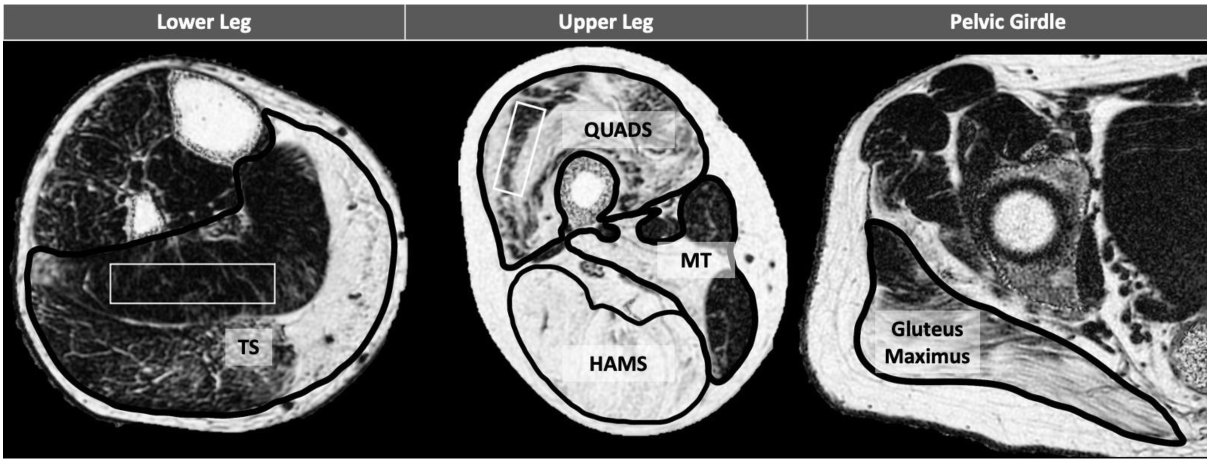

All MRI and MRS data were processed at a central reading laboratory (University of Florida, Gainesville, FL, USA). Fat fraction maps were generated, and the borders of the quadriceps group (vastus lateralis, vastus medialis, vastus intermedius, rectus femoris), hamstrings group (biceps femoris, semitendinosus, semimembranosus), medial compartment of the thigh (adductor magnus, adductor longus, gracilis, sartorius), triceps surae group (soleus and gastrocnemius), and gluteus maximus muscle were manually traced on three contiguous slices (landmark and the two slices immediately distal to it) using custom-written software with mean values reported for each muscle (3). MRS fat fraction was assessed using area integration of the phase-corrected spectra from the lipid and water region of the spectrum, with correction for relaxation effects as previously described (3). For the whole thigh group, CSA values were calculated as the sum of the values in the quadriceps, hamstrings and medial thigh groups; fat fraction values were the average of the values in these three groups.

Initial dose administered

Supplementary Table 1. Initial givinostat and placebo dose administered.

	Weight (kg)				
	**≥30–<40**	**≥40–<50**	**≥50–<60**	**≥60–<70**	**≥70**
**High dose**					
Dose (mg) bid	40	50	55	60	70
Oral suspension (mL) bid	4.0	5.0	5.5	6.0	7.0
**Low dose**					
Dose (mg) bid	26.7	33.3	36.7	40	46.7
Oral suspension (mL) bid	2.7	3.3	3.7	4.0	4.7

bid, twice daily

Safety stopping rules

Study treatment had to be permanently discontinued if any of the following events occurred:

- severe drug-related diarrhea (increase of ≥7 stools per day)
- any drug-related serious adverse event
- QTcF >500 msec based on average QTc value of triplicate electrocardiograms
- platelet count ≤50 x 10^9/L
- white blood cell count ≤2.0 x 10^9/L
- hemoglobin ≤ 8.0 g/dL.

Study treatment had to be temporarily interrupted if any of the following events occurred:

- moderate or severe diarrhea (more than 4 stools per day)
- platelet count <75 x 10^9/L but >50 x 10^9/L
- white blood cell count <3.0 x 10^9/L but >2.0 x 10^9/L
- hemoglobin <10.0 g/dL but >8.0 g/dL
- triglycerides >300mg/dL.

Outcomes

The histology parameters were determined from biopsy of the brachial biceps, using the mean of available fields (fiber size variability was calculated as the median of the

interquartile range of the total CSA). Dystrophin values were the percentage compared to the arithmetic mean of two positive control (i.e., healthy subjects) bands present in the same western blot (see supplement).

Chemical shift-encoded MRI was used to measure fat fraction in the whole thigh, quadriceps, medial thigh, hamstrings, triceps surae, and gluteus maximus. Fat fraction for each compartment was calculated as the average of all pixels on the three slices, and whole-thigh fat fraction was calculated as the average fat fraction of the quadriceps, hamstrings, and medial thigh compartments. MRS measures of fat fraction in the vastus lateralis and soleus were determined using automated processes.

Each item of the MFM was scored on a four-point Likert scale (0, cannot initiate the task or maintain the starting position; 3, performs the task fully and without compensation strategies). Three measurements from each muscle group on each side were recorded for the knee extension and elbow flexion assessments, with the mean of these three measurements used for all analyses. The rise from floor velocity was calculated as 1/time in sec taken to stand up from a sitting position on the floor with legs outstretched, with the stair climb velocity calculated as 4/time in sec taken to climb four standard stairs, and the walk/run velocity as 10/time in sec taken to run/walk 10 m. Velocity was scored as 0 for patients unable to perform a task.

Results

Supplementary Table 2. Western blot analysis at baseline and Month 12.

Endpoint<br>Treatment group	Baseline, mean (SD)	Month 12	
		**Change from baseline, LSM (95% CI)**	**Givinostat–placebo difference, LSM (95% CI); p value**
**Western Blot dystrophin 1**			
Givinostat	31.8 (16.07)	1.33 (1.06, 1.66)	1.00 (0.82, 1.23); 0.9691
Placebo	40.5 (19.16)	1.32 (1.00, 1.74)	
**Western Blot dystrophin 2**			
Givinostat	18.9 (11.51)	0.87 (0.58, 1.31)	0.73 (0.54, 0.99); 0.0427
Placebo	20.4 (19.46)	1.19 (0.74, 1.92)	

LSM, least squares mean; CSA, cross-sectional area. Dystrophin 1: dystrophin Rod domain antibody; Dystrophin 2: dystrophin C-terminus antibody.

Supplementary Table 3. MRI cross-sectional and contractile areas at baseline and Month 12.

Endpoint<br>Treatment group	Baseline, mean (SD)	Month 12	
		Change from baseline, LSM (95% CI)	Givinostat–placebo difference, LSM (95% CI); p value
**MRI – cross-sectional area, cm^2**			
**Whole thigh**			
Givinostat	84.7 (30.18)	2.78 (0.15, 5.41)	0.40 (–2.45, 3.26); 0.7770
Placebo	83.1 (20.61)	2.38 (–0.82, 5.58)	
**Quadriceps**			
Givinostat	35.4 (13.64)	1.00 (–0.17, 2.16)	–0.09 (–1.35, 1.18); 0.8926
Placebo	32.7 (8.77)	1.08 (–0.33, 2.49)	
**Medial thigh**			
Givinostat	25.8 (11.79)	0.67 (–0.47, 1.81)	0.35 (–0.88, 1.58); 0.5720
Placebo	25.8 (8.20)	0.32 (–1.06, 1.70)	
**Hamstrings**			
Givinostat	23.5 (8.54)	1.10 (0.11, 2.10)	0.11 (–0.97, 1.18); 0.8386
Placebo	24.7 (6.29)	0.99 (–0.21, 2.20)	
**Triceps surae**			
Givinostat	51.8 (18.41)	–0.03 (–3.70, 3.63)	–0.22 (–2.68, 2.25); 0.8591
Placebo	62.9 (14.82)	0.18 (–3.76, 4.12)	
**Gluteus maximus**			
Givinostat	39.1 (13.36)	1.28 (–0.49, 3.06)	0.32 (–1.60, 2.24); 0.7392
Placebo	40.1 (12.39)	0.97 (–1.19, 3.12)	
**MRI – contractile area, cm^2**			
**Whole thigh**			
Givinostat	37.0 (15.23)	0.31 (–0.85, 1.47)	1.37 (0.08, 2.65); 0.0375
Placebo	30.0 (11.53)	–1.06 (–2.47, 0.35)	
**Quadriceps**			
Givinostat	15.8 (8.47)	0.14 (–0.46, 0.74)	0.63 (–0.01, 1.27); 0.0528
Placebo	12.5 (4.57)	–0.49 (–1.20, 0.21)	
**Medial thigh**			
Givinostat	13.3 (5.95)	0.16 (–0.34, 0.67)	0.36 (–0.20, 0.91); 0.2012
Placebo	11.0 (5.04)	–0.20 (–0.81, 0.42)	
**Hamstrings**			
Givinostat	7.9 (6.15)	1.03 (0.96, 1.10)	1.05 (0.98, 1.13); 0.1939
Placebo	6.5 (4.98)	0.98 (0.90, 1.06)	

Endpoint<br>Treatment group	Baseline, mean (SD)	Month 12<br>Change from baseline, LSM (95% CI)	Month 12<br>Givinostat–placebo difference, LSM (95% CI); p value
**Triceps surae**			
Givinostat	33.3 (12.26)	–0.54 (–3.60, 2.51)	0.75 (–1.33, 2.83); 0.4676
Placebo	39.7 (12.09)	–1.29 (–4.63, 2.05)	
**Gluteus maximus**			
Givinostat	14.6 (7.94)	0.26 (–0.42, 0.93)	0.54 (–0.21, 1.30); 0.1549
Placebo	11.1 (3.04)	–0.29 (–1.13, 0.56)	

MRI, magnetic resonance imaging; LSM, least squares mean.

Supplementary Table 4. Hand-held myometry endpoints at baseline and Month 12.

Endpoint<br>Treatment group	Baseline, mean (SD)	Month 12	
		**Change from baseline, LSM (95% CI)**	**Givinostat–placebo difference, LSM (95% CI); p value**
**Left knee extension**			
Givinostat	60.7 (40.14)	–1.48 (–10.21, 7.25)	3.57 (–7.27, 14.41); 0.5099
Placebo	43.9 (20.35)	–5.05 (–15.95, 5.85)	
**Right knee extension**			
Givinostat	60.2 (41.08)	0.70 (–5.21, 6.62)	1.16 (–5.73, 8.06); 0.7355
Placebo	39.6 (17.32)	–0.46 (–7.71, 6.78)	
**Left elbow flexion**			
Givinostat	95.8 (51.48)	1.55 (–10.05, 13.15)	3.92 (–11.22, 19.06); 0.6037
Placebo	81.8 (55.07)	–2.37 (–17.49, 12.74)	
**Right elbow flexion**			
Givinostat	97.3 (55.63)	–1.28 (–13.38, 10.83)	4.10 (–12.35, 20.55); 0.6178
Placebo	80.1 (59.56)	–5.38 (–21.27, 10.51)	

LSM, least squares mean.

Supplementary Table 5. Platelet counts and triglyceride levels at baseline, Month 12, and minimum value recorded over the study.

	Givinostat (N=34)	Placebo (N=17)
**Platelet counts**		
Mean (SD); range, 10^9 cells per L		
Baseline	267.0 (47.54); 114 to 344	281.1 (93.71); 172 to 568
Month 12	205.8 (58.06); 133 to 332	282.9 (89.83); 165 to 566
Change from baseline at Month 12	–61.2 (58.29); –168 to 72	1.8 (27.34); –62 to 47
Minimum over the study	132.3 (43.36); 64 to 237	248.9 (79.99); 152 to 493
Shift from normal at baseline to low at any time during the study, n (%)	12 (36.4)	0
Patients who reduced dose due to platelet count decrease	18 (52.9)	0
**Triglyceride levels**		
Mean (SD); range, mmol/L		
Baseline	1.39 (0.697); 0.8 to 4.3	1.58 (0.657); 0.7 to 2.8
Month 12	1.93 (0.875); 0.6 to 3.9	1.53 (0.634); 0.5 to 2.4
Change from baseline at Month 12	0.52 (0.726); –0.7 to 2.2	–0.06 (0.465); –1.0 to 0.8
Shift, to high at any time during the study, n (%)		
From normal at baseline	17 (50.0)	4 (23.5)
From high at baseline	2 (5.9)	3 (17.6)

References

1. Peverelli L, Testolin S, Villa L, D'Amico A, Petrini S, Favero C, Magri F, Morandi L, Mora M, Mongini T, et al. Histologic muscular history in steroid-treated and untreated patients with Duchenne dystrophy. *Neurology* (2015) 85:1886–1893. doi: 10.1212/WNL.0000000000002147

2. Hu HH, Yokoo T, Bashir MR, Sirlin CB, Hernando D, Malyarenko D, Chenevert TL, Smith MA, Serai SD, Middleton MS, et al. Linearity and bias of proton density fat fraction as a quantitative imaging biomarker: A multicenter, multiplatform, multivendor phantom study. *Radiology* (2021) 298:640–651. doi: 10.1148/radiol.2021202912

3. Triplett WT, Baligand C, Forbes SC, Willcocks RJ, Lott DJ, DeVos S, Pollaro J, Rooney WD, Sweeney HL, Bönnemann CG, et al. Chemical shift-based MRI to measure fat fractions in dystrophic skeletal muscle. *Magn Reson Med* (2014) 72:8–19. doi: 10.1002/mrm.24917

4. Forbes SC, Willcocks RJ, Triplett WT, Rooney WD, Lott DJ, Wang DJ, Pollaro J, Senesac CR, Daniels MJ, Finkel RS, et al. Magnetic resonance imaging and spectroscopy assessment of lower extremity skeletal muscles in boys with Duchenne muscular dystrophy: a multicenter cross sectional study. *PLoS One* (2014) 9:e106435. doi: 10.1371/JOURNAL.PONE.0106435
